# The Doors and People Test: The Effect of Frontal Lobe Lesions on Recall and Recognition Memory Performance

**DOI:** 10.1037/neu0000240

**Published:** 2016-01-11

**Authors:** Sarah E. MacPherson, Martha S. Turner, Marco Bozzali, Lisa Cipolotti, Tim Shallice

**Affiliations:** 1 Institute of Cognitive Neuroscience, University College London and Department of Neuropsychology, National Hospital for Neurology and Neurosurgery, London, United Kingdom; 2 Wellcome Department of Imaging Neuroscience, University College London and Neuroimaging Laboratory, Santa Lucia Foundation, IRCCS, Rome, Italy; 3 Department of Neuropsychology, National Hospital for Neurology and Neurosurgery and Dipartimento di Psicologia, University of Palermo; 4 Institute of Cognitive Neuroscience, University College London and SISSA, Trieste, Italy

**Keywords:** frontal lobes, episodic memory, recall, recognition

## Abstract

***Objective:*** Memory deficits in patients with frontal lobe lesions are most apparent on free recall tasks that require the selection, initiation, and implementation of retrieval strategies. The effect of frontal lesions on recognition memory performance is less clear with some studies reporting recognition memory impairments but others not. The majority of these studies do not directly compare recall and recognition within the same group of frontal patients, assessing only recall or recognition memory performance. Other studies that do compare recall and recognition in the same frontal group do not consider recall or recognition tests that are comparable for difficulty. Recognition memory impairments may not be reported because recognition memory tasks are less demanding. ***Method:*** This study aimed to investigate recall and recognition impairments in the same group of 47 frontal patients and 78 healthy controls. The Doors and People Test was administered as a neuropsychological test of memory as it assesses both verbal and visual recall and recognition using subtests that are matched for difficulty. ***Results:*** Significant verbal and visual recall and recognition impairments were found in the frontal patients. ***Conclusion:*** These results demonstrate that when frontal patients are assessed on recall and recognition memory tests of comparable difficulty, memory impairments are found on both types of episodic memory test.

It is widely accepted that the frontal lobes play an important role in memory. With certain exceptions such as confabulation (Turner, Cipolotti, Yousry, & Shallice, 2008), memory impairments in frontal patients are generally thought to be secondary to impairments in executive processes (e.g., planning, inhibition, monitoring) rather than a memory deficit per se (Kopelman, 2002). In particular, the frontal lobes are thought to contribute to the monitoring and control of memory processes during encoding and retrieval (Kapur et al., 1995; Moscovitch & Winocur, 1995; Shallice et al., 1994). In a meta-analysis of frontal patients’ memory performance, Wheeler, Stuss, and Tulving (1995) found that frontal patients were impaired in 80% of studies involving free recall tests, 50% of studies involving cued-recall tests but only 8% of studies involving recognition memory tests. The greater frontal impairment on free recall compared to cued recall and recognition memory was explained in terms of free recall placing greater demands on strategic retrieval than cued recall or recognition memory.

Early studies of recognition memory reported that frontal patients are not impaired (e.g., Janowsky, Shimamura, Kritchevsky, & Squire, 1989; Milner, Corsi, & Leonard, 1991). For example, Janowsky et al. (1989) reported no impairment in recognition memory performance in a small group of patients with frontal lesions due to an abscess, cerebrovascular accident, or traumatic brain injury. Impairments were also not found in a larger group of 59 patients who underwent excisions in either the frontal or frontotemporal lobes for the relief of epilepsy (Milner et al., 1991). However, some frontal patients have demonstrated impairments on recognition memory tasks, often due to a tendency to produce a higher number of false alarms (e.g., Alexander, Stuss, & Fansabedian, 2003; Baldo, Delis, Kramer, & Shimamura, 2002; Delbecq-Derouesné, Beauvois, & Shallice, 1990; MacPherson et al., 2008). For instance, Baldo et al. (2002) demonstrated difficulties in differentiating between targets and distractors on a verbal recognition task in a small number of 11 patients with lesions due to infarction, aneurysm rupture, arteriovenous malformation, cyst, or meningioma. In studies where frontal patients have been subdivided into frontal subgroups, a high rate of false alarms has been reported in left dorsolateral prefrontal patients (Alexander et al., 2003). Generally, though, impairments in recognition memory performance in frontal patients tend to be reported less consistently than deficits in free recall performance.

Few patient studies have, however, allowed comparison of recall and recognition performance in the same frontal lobe patients. Those few studies that have compared recall and recognition have reported inconsistent findings. Some studies have reported impairments in both recall and recognition memory (e.g., Alexander et al., 2003; Baldo et al., 2002; Kopelman & Stanhope, 1998), whereas others have demonstrated disproportionate impairments in free recall (e.g., Janowsky et al., 1989; Shimamura, Janowsky, & Squire, 1991) or recognition (e.g., Delbecq-Derouesne et al., 1990; Schacter, Curran, Galluccio, Milberg, & Bates, 1996). It should be noted that in some of these studies recognition memory performance has been confounded by ceiling effects (Janowsky et al., 1989). They also differ in their design with some studies using a multiple-alternative forced-choice design but others using single-item decisions to targets and lures. Wheeler and colleagues’ meta-analysis included recognition memory data from studies adopting both these designs.

The Doors and People Test was devised by Baddeley, Emslie, and Nimmo-Smith (1994) for the purpose of directly comparing recall and recognition performance within the same test. It includes four subtests assessing recall and recognition for both verbal and visual information, which are thought to be comparable in terms of difficulty (e.g., Mayes, Holdstock, Isaac, Hunkin, & Roberts, 2002). Chapman and Chapman (1973) argued for the importance of matching tests used to assess individuals with neurological disorders for mean difficulty to ensure poor performance refers to a specific impairment rather than simply being an artifact. Moreover, standardized scores are available for all subtests so one can use the variability of the normal population to assess the degree of impairment on the different subtests. A previous neuropsychological study investigating specialization within the medial temporal lobes when performing the Doors and People Test reported poor verbal recall and recognition memory performance associated with left medial temporal lobe lesions and poor visual recall and recognition memory performance associated with right medial temporal lobe lesions (Morris, Abrahams, Baddeley, & Polkey, 1995). To the best of our knowledge, no studies so far have investigated frontal patients’ performance on the different subtests of the Doors and People Test.

## Method

### Participants

The patient group was recruited from the National Hospital for Neurology and Neurosurgery and consisted of 47 frontal patients (27 men, 20 women), of whom 41 were right-handed. No patients had any other significant neurological or psychiatric disorders. Patients were excluded if they had received radiotherapy or chemotherapy over the previous 4 weeks. Antiepileptic drugs were allowed, but all patients had to have taken their current medication for at least 2 months to reduce the risk of any side effects impacting upon their neuropsychological performance. The frontal lesions were classified by a neurologist (Marco Bozzali) on the basis of MRI scans (or CT scans where MRI was unavailable). They had to show a focal, unilateral lesion of the frontal lobes, in the absence of any remarkable abnormality in the rest of the brain. The etiologies included tumors/abscesses: glioma = 20; meningioma = 10; abscess = 1; and lesions due to vascular malformations and/or bleeding: anterior communicating aneurysm = 12; subarachnoid hemorrhage of unknown etiology = 2; cerebral cavernous malformation = 1; arteriovenous malformation = 1. Importantly, it has previously been documented that there is no significant difference in the performance of high or low grade tumor, meningioma or stroke on a series of executive, naming and perception tests. This suggested that grouping together patients with different etiologies is methodologically justifiable (Cipolotti et al., 2015). The time since lesion onset/surgery was 12.93 months (*SD* = 23.77).

Patient performance was compared with 78 healthy controls (40 men, 38 women), who had no previous history of head injury or stroke, major neurological or psychiatric illness, or alcohol abuse. Sixty-eight were right-handed. The frontal patients and the healthy controls did not differ significantly in terms of age (*M* = 46.06, *SD* = 13.31 and *M* = 48.83, *SD* = 13.44, respectively, *p* = .31) or years of education (*M* = 13.51, *SD* = 3.28 and *M* = 13.44, *SD* = 3.06, respectively, *p* = .90). All participants were native English speakers. The study was approved by the National Hospital for Neurology and Neurosurgery & Institute of Neurology Joint Research Ethics Committee. Consent was obtained according to the Declaration of Helsinki.

### Procedure

#### Background neuropsychological measures

All patients and controls performed the National Adult Reading Test—Revised to estimate premorbid levels of functioning (Nelson & Willison, 1991) and Raven’s Progressive Matrices to assess nonverbal abstract reasoning (Raven, Raven, & Court, 1998). The Graded Naming Test (McKenna & Warrington, 1983) assessed naming abilities and the Fragmented Letters subtest from the Visual Object and Space Perception Battery (Warrington & James, 1991) assessed perceptual abilities. Executive abilities were assessed in terms of phonemic fluency using the Controlled Oral Word Association (letters F, A, and S; Spreen & Strauss, 1998) and inhibition using the Stroop Test (Trenerry, Crosson, Deboe, & Leber, 1989).

#### Doors and People Test

The Doors and People Test was administered according to the procedure described in the manual (Baddeley et al., 1994). The subtests were administered in the following order: immediate verbal recall, visual recognition, delayed verbal recall, immediate visual recall, verbal recognition, and delayed visual recall. Both recognition memory tasks adopt a multiple-alternative forced-choice design.

##### Verbal recall (People subtest)

Photographs of four characters with their names and occupations printed below are presented for 3 seconds each and the character’s name and occupation is read aloud (e.g., “This is the minister. His name is Cuthbert Cattermole”). Immediately after all four pictures are presented, participants are given each character’s occupation and asked to recall the associated name (e.g., “What is the name of the minister?”). This procedure is repeated until all four names are correctly recalled (a maximum of three times). One point is awarded for each first name and surname correctly recalled with an additional mark for each correct pairing (maximum out of 36) and an age-scaled score is obtained. Lastly, delayed recall of the names was assessed after administration of the visual recognition subtest. A verbal forgetting score is derived from the difference between the scores on the final trial on the immediate recall subtest and the delayed recall subtest.

##### Visual recall (Shapes subtest)

Four simple line drawings of crosses are presented for 5 s each and participants are asked to copy them. Participants are then asked to immediately draw the four shapes from memory. The procedure is repeated until all four shapes are recalled correctly (a maximum of 3 times). However, the shapes are not copied for the second and third trials. Finally, delayed recall of the shapes was assessed after the verbal recognition subtest. Each drawing is awarded a maximum of 3 points and an age-scaled score is obtained from the total score for the three trials (out of 36). A delayed visual recall age-scaled score is derived from the maximum score of 12.

##### Verbal recognition (Names subtest)

In part A of the Names subtest, 12 female names (both the forename and surname) are shown for 3 s each (e.g., Diane Neeson), and participants are asked to read the names out loud. Participants are immediately presented with 12 lists of four names (the target name together with three distracter names) and asked to choose the target name. For part B, this procedure is then repeated with 12 male names. One point is awarded for each correct answer giving a total score out of 24 and then an age-scaled score is derived.

##### Visual recognition (Doors subtest)

In parts A and B of the Doors subtest, participants are presented with 12 colored photographs of different types of doors for 3 s each. The same target doors are then presented immediately afterward in a 2 × 2 array together with three distracter doors from the same category (e.g., a stable door) and participants are asked to identify the target door. In part A, the distracters are different categories of door to the target door (e.g., a garage door, a French door, a front door) whereas in part B, the distracters are the same type of door (e.g., all stable doors). One point is awarded for each correct response and the combined A and B scores (out of 24) are used to obtain an age-scaled score.

### Statistical Analysis

Shapiro-Wilks tests demonstrated that the data for the frontal patients and the healthy control group on the background neuropsychological tests and the subtests from the Doors and People Test were often not normally distributed (*p* ≤ .02). Bootstrap analyses were therefore conducted as they require fewer assumptions than traditional parametric tests and are desirable when control data are positively skewed (Delucchi & Bostrom, 2004). One thousand bootstrap resamples were created by randomly resampling with replacement from the original data. A 95% confidence interval (CI) for the mean is estimated using these 1,000 bootstrap samples. The 1,000 resamples were then examined using independent samples *t* tests to compare the performance of the frontal and control groups on each of the background neuropsychological and experimental tests. In addition, frontal patients with tumors/abscesses (*n* = 32) or vascular lesions (*n* = 15) were compared using independent samples *t* tests.

## Results

### Background Neuropsychological Measures

Table 1 shows the means, standard deviations, and minimum and maximum values for the frontal patients and healthy controls on the background neuropsychological measures. The frontal group were significantly less accurate at naming on the Graded Naming Test compared to the healthy controls, *t*(76.25) = −2.38, *p* < .05 (95% CI = −4.07 to −0.38). On the executive measures, the frontal group produced significantly fewer words on fluency compared to controls, *t*(123) = −5.84, *p* < .005 (95% CI = −20.52 to −10.42), and were significantly slower than the controls on Stroop Color-Word time, *t*(41.28) = 2.52, *p* < .05 (95% CI = 9.22 to 57.81). The frontal and control groups did not significantly differ in performance on the National Adult Reading Test—Revised (*p* = .10), Raven’s APM (*p* = .43), fluency errors (*p* = .73), Stroop Color-Word errors (*p* = .13), or the Fragmented Letters test (*p* = .58).[Table-anchor tbl1]

### Doors and People Test

For each subtest, the mean raw score that gave rise to a scaled score of 10 (average performance) over the five age groups in the Doors and People Test manual was divided by the subtest’s maximum score. This led to the following percentage difficulty scores for each subtest: verbal recall = 71.67%; verbal recognition = 71.67%; visual recall = 82.78% and visual recognition = 74.17%. The percentage difficulty scores suggest that while visual recall is possibly slightly easier than the other subtests, the authors of the Doors and People Test made a reasonable attempt to match the subtests for difficulty.

Performance on the Doors and People Test was converted into age-scaled scores to allow for direct comparison across subtests (see Table 2). Analysis of the overall recall score demonstrated that the frontal group recalled significantly fewer items than the control group (*M* = 8.77, *SD* = 3.37; *M* = 11.35, *SD* = 3.24, respectively), *t*(123) = −4.25, *p* < .005 (95% CI = −3.87 to −1.32). Frontal patients also recognized significantly fewer items than controls when the overall recognition scores were compared (*M* = 9.60, *SD* = 3.27; *M* = 11.47, *SD* = 3.19, respectively), *t*(123) = −3.16, *p* < .005 (95% CI = −3.06 to −0.66).[Table-anchor tbl2]

#### Verbal recall (People subtest)

An independent samples *t* test revealed that the frontal group recalled significantly fewer names than the controls on immediate verbal recall, *t*(123) = −3.59, *p* < .0001. There was significantly more verbal forgetting in the frontal group compared with controls, *t*(77.90) = −3.59, *p* < .005. However, tumor/abscess and vascular patients did not significantly differ on either measure (*p* > .31).

#### Visual recall (Shapes subtest)

The frontal patients recalled significantly fewer items than the controls on immediate visual recall, *t*(123) = −3.50, *p* < .005, but there was no significant difference between the frontal and control groups in terms of visual forgetting (*p* = .93). The frontal subgroups did not significantly differ (*p* > .18).

#### Verbal recognition (Names subtest)

The frontal patients recognized significantly fewer names than the healthy controls on verbal recognition, *t*(123) = −2.49, *p* < .05. However, the frontal subgroups did not significantly differ (*p* = .70).

#### Visual recognition (Doors subtest)

On visual recognition, the frontal patients performed significantly more poorly than the healthy controls, *t*(123) = −2.72, *p* < .01. The frontal subgroups did not significantly differ (*p* = .57).

The effect sizes (Cohen’s *d*) for the frontal patients were calculated (verbal recall: *M* = −0.66, *SD* = 1.03; visual recall: *M* = −0.69, *SD* = 1.16; verbal recognition: *M* = −0.47, *SD* = 1.05; visual recognition: *M* = −0.49, *SD* = 0.92). A repeated measures analyses of variance revealed no significant effect of memory measure on performance (*p* = .16). This result suggests that the size of the difference between the frontal patients and healthy controls’ performance did not significantly differ across the verbal and visual recall and recognition memory measures.

To determine whether performance on the more difficult part B was driving the frontal effect on the recognition memory tasks, separate 2 (frontal vs. healthy control) × 2 (part A vs. part B) analyses of variance were conducted on the age-scaled scores for parts A and B (see Table 2). For the Names subtest, there was a significant main effect of group, *F*(1, 119) = 8.54, *p* < .005, where frontal patients performed significantly more poorly than the healthy controls. There was not a significant main effect of condition (*p* = .65) or a two-way interaction (*p* = .88). For the Doors subtest, again there was a significant main effect of group, *F*(1, 118) = 7.01, *p* < .01, where frontal patients achieved significantly lower scores than the healthy controls. There was not a significant main effect of condition (*p* = .20) or two-way interaction (*p* = .70). The lack of a significant interaction on both recognition memory tests suggests that frontal patients were poorer than controls in terms of recognition memory performance on both the easy and difficult versions of the tasks.

## Discussion

In the present study, the recall and recognition subtests of the Doors and People Test (Baddeley et al., 1994) were administered to frontal patients. The main aim of the study was to examine performance on tests of recall and recognition thought to be comparable in terms of difficulty within the same group of frontal patients. The frontal patients were significantly impaired on both the recall and recognition subtests compared to healthy controls. However, although not statistically different, the effect sizes for recall in frontal patients were directionally larger than for recognition memory.

The People subtest of the Doors and People Test assesses verbal recall by providing participants with the occupation of each person to be recalled at test (e.g., What is the name of the minister?). Other studies have tended to provide word stems to aid the recall of study items (e.g., Kopelman & Stanhope, 1997) or category labels (e.g., Incisa della Rocchetta & Milner, 1993). Although the lesion studies adopting word stems at recall have tended not to find frontal effects, our frontal group was impaired on the People subtest when names had to be recalled.

In terms of verbal and visual forgetting, which compare immediate and delayed recall, the frontal patients showed a significant impairment in verbal forgetting when compared with healthy controls. In contrast, our frontal group was not impaired in visual forgetting. This dissociation between verbal and visual forgetting suggests that there may be fundamental differences in the way individuals perform these two tasks. Verbal recall, in particular, is likely to require semantic organizational strategies to assist learning of the associations between names and occupations. Such associations require the production of a mediating link or pathway to facilitate the appropriate response (i.e., occupation) when presented with a particular stimulus (e.g., name), a process known to involve the frontal cortex (Fletcher, Shallice, & Dolan, 2000). The lack of a mediator would make the patients much more susceptible to forgetting. On the other hand, the visual recall of a series of lines in the Shapes subtest is unlikely to rely on such strategies.

Of course, there some inevitable complications with any study assessing large numbers of frontal patients (e.g., different aetiologies, white matter abnormalities). However, no significant difference was found between our tumor and vascular patients. The two important conclusions from our study are that recognition memory is impaired in frontal patients, but recall is more impaired than recognition. These findings are in line with the pattern of effects reported by Wheeler et al. (1995). Our data suggest that when a large sample of frontal patients is assessed with tests of comparable difficulty, the memory impairment is present both in recognition and recall.

## Figures and Tables

**Table 1 tbl1:** Performance of the Patient Groups and Healthy Controls on the Background Measures

Group	NART IQ	Raven’s APM (/12)	GNT (/30)	Phonemic fluency total words	Phonemic fluency errors	Stroop CW time (s)	Stroop CW errors	Frag. letters (/20)
Frontal	108.45 (13.08)	8.30 (2.42)	20.21* (5.68)	32.34** (14.83)	1.80 (2.02)	160.47* (72.73)	2.23 (5.56)	19.45 (.93)
	68–129	1–12	7–30	5–65	0–7	70–420	0–28	15–20
Healthy controls	112.13 (9.69)	8.79 (2.10)	22.49 (4.19)	47.76 (13.37)	1.68 (1.62)	128.64 (31.35)	.82 (1.75)	19.36 (.81)
	85–129	3–12	9–30	17–82	0–6	62–420	0–9	16–20
*Note*. NART = National Adult Reading Test; APM = Advanced Progressive Matrices; GNT = Graded Naming Test; CW = Colour-Word; Frag. letters = Fragmented letters.								
* *p* < .05. ** *p* < .005.								

**Table 2 tbl2:** Mean Age-Scaled Scores and Standard Deviations for the Frontal Patients and Controls on the Doors and People Test

	Frontal patients		Healthy controls		Bootstrap 95% confidence intervals		
	*M*	*SD*	*M*	*SD*	Mean difference	Lower, upper	*P* value
Verbal recall	8.53	3.72	10.95	3.60	−2.42	[−3.73, −1.02]	<.005
Verbal forgetting	8.85	2.96	10.64	2.25	−1.79	[−2.71, −.82]	<.005
Visual recall	9.57	3.03	11.37	2.62	−1.80	[−2.78, −.68]	<.005
Visual forgetting	10.38	1.57	10.41	1.76	−.03	[−.63, .57]	.31
Verbal recognition	10.11	3.67	11.74	3.50	−1.64	[−2.93, −.33]	<.05
Part A^a^	9.82	3.62	11.38	2.95			
Part B^a^	10.02	3.91	11.53	3.26			
Visual recognition	9.19	2.67	10.60	2.89	−1.41	[−2.37, −.40]	<.01
Part A^b^	9.73	3.07	10.85	2.59			
Part B^b^	9.26	3.00	10.58	3.32			
^a^ The scores of 44 frontal patients (data were no longer available for three patients) and 77 healthy controls (data were no longer available for one control). ^b^ The scores of 43 frontal patients (data were no longer available for four patients) and 77 healthy controls (data were no longer available for one control).							

## References

[c1] Alexander M. P., Stuss D. T., & Fansabedian N. (2003). California Verbal Learning Test: Performance by patients with focal frontal and non-frontal lesions. Brain: A Journal of Neurology, 126, 1493–1503. 10.1093/brain/awg128 12764068

[c2] Baddeley A. D., Emslie H., & Nimmo-Smith I. (1994). The Doors and People Test: A test of visual and verbal recall and recognition. Bury-St-Edmunds, UK: Thames Valley Test Company.

[c3] Baldo J. V., Delis D., Kramer J., & Shimamura A. P. (2002). Memory performance on the California Verbal Learning Test-II: Findings from patients with focal frontal lesions. Journal of the International Neuropsychological Society, 8, 539–546. 10.1017/S135561770281428X 12030307

[c4] Chapman L. J., & Chapman J. P. (1973). Problems in the measurement of cognitive deficit. Psychological Bulletin, 79, 380–385. 10.1037/h0034541 4707457

[c5] Cipolotti L., Healy C., Chan E., Bolsover F., Lecce F., White M., . . . Bozzali M. (2015). The impact of different aetiologies on the cognitive performance of frontal patients. Neuropsychologia, 68, 21–30. 10.1016/j.neuropsychologia.2014.12.025 25556811 PMC4410793

[c6] Delbecq-Derouesné J., Beauvois M. F., & Shallice T. (1990). Preserved recall versus impaired recognition. A case study. Brain: A Journal of Neurology, 113, 1045–1074. 10.1093/brain/113.4.1045 2397382

[c7] Delucchi K. L., & Bostrom A. (2004). Methods for analysis of skewed data distributions in psychiatric clinical studies: Working with many zero values. The American Journal of Psychiatry, 161, 1159–1168. 10.1176/appi.ajp.161.7.1159 15229044

[c8] Fletcher P. C., Shallice T., & Dolan R. J. (2000). “Sculpting the response space”—an account of left prefrontal activation at encoding. NeuroImage, 12, 404–417. 10.1006/nimg.2000.0633 10988034

[c9] Incisa della Rocchetta A., & Milner B. (1993). Strategic search and retrieval inhibition: The role of the frontal lobes. Neuropsychologia, 31, 503–524. 10.1016/0028-3932(93)90049-6 8341411

[c10] Janowsky J. S., Shimamura A. P., Kritchevsky M., & Squire L. R. (1989). Cognitive impairment following frontal lobe damage and its relevance to human amnesia. Behavioral Neuroscience, 103, 548–560. 10.1037/0735-7044.103.3.548 2736069

[c11] Kapur S., Craik F. I. M., Jones C., Brown G. M., Houle S., & Tulving E. (1995). Functional role of the prefrontal cortex in retrieval of memories: A PET study. Neuroreport: An International Journal for the Rapid Communication of Research in Neuroscience, 6, 1880–1884. 10.1097/00001756-199510020-00014 8547589

[c12] Kopelman M. D. (2002). Disorders of memory. Brain: A Journal of Neurology, 125, 2152–2190. 10.1093/brain/awf229 12244076

[c13] Kopelman M. D., & Stanhope N. (1997). Rates of forgetting in organic amnesia following temporal lobe, diencephalic, or frontal lobe lesions. Neuropsychology, 11, 343–356. 10.1037/0894-4105.11.3.343 9223139

[c14] Kopelman M. D., & Stanhope N. (1998). Recall and recognition memory in patients with focal frontal, temporal lobe and diencephalic lesions. Neuropsychologia, 36, 785–796. 10.1016/S0028-3932(97)00167-X 9751442

[c15] MacPherson S. E., Bozzali M., Cipolotti L., Dolan R. J., Rees J. H., & Shallice T. (2008). Effect of frontal lobe lesions on the recollection and familiarity components of recognition memory. Neuropsychologia, 46, 3124–3132. 10.1016/j.neuropsychologia.2008.07.003 18675284 PMC2666796

[c16] Mayes A. R., Holdstock J. S., Isaac C. L., Hunkin N. M., & Roberts N. (2002). Relative sparing of item recognition memory in a patient with adult-onset damage limited to the hippocampus. Hippocampus, 12, 325–340. 10.1002/hipo.1111 12099484

[c17] McKenna P., & Warrington E. K. (1983). Graded Naming Test. Windsor, UK: NFER Nelson.

[c18] Milner B., Corsi P., & Leonard G. (1991). Frontal-lobe contribution to recency judgements. Neuropsychologia, 29, 601–618. 10.1016/0028-3932(91)90013-X 1944864

[c19] Morris R. G., Abrahams S., Baddeley A. D., & Polkey C. E. (1995). Doors and people: Visual and verbal memory after unilateral temporal lobectomy. Neuropsychology, 9, 464–469. 10.1037/0894-4105.9.4.464

[c20] Moscovitch M., & Winocur G. (1995). Frontal lobes, memory, and aging In Grafman J., Holyoak K. J., & Boller B. (Eds.), Structure and functions of the human prefrontal cortex (pp. 119–150). New York, NY: The New York Academy of Sciences. 10.1111/j.1749-6632.1995.tb38135.x 8595020

[c21] Nelson H. E., & Willison J. (1991). National Adult Reading Test (NART): Test Manual (2nd ed.). Windsor, UK: NFER Nelson.

[c22] Raven J., Raven J. C., & Court J. H. (1998). Manual for Raven’s Progressive Matrices and Vocabulary Scales. Oxford, UK: Oxford Psychologists Press.

[c23] Schacter D. L., Curran T., Galluccio L., Milberg W. P., & Bates J. F. (1996). False recognition and the right frontal lobe: A case study. Neuropsychologia, 34, 793–808. 10.1016/0028-3932(95)00165-4 8817509

[c24] Shallice T., Fletcher P., Frith C. D., Grasby P., Frackowiak R. S., & Dolan R. J. (1994). Brain regions associated with acquisition and retrieval of verbal episodic memory. Nature, 368, 633–635. 10.1038/368633a0 8145849

[c25] Shimamura A. P., Janowsky J. S., & Squire L. R. (1991). What is the role of frontal lobe damage in memory disorders? In Levin H. D., Eisenberg H. M., & Benton A. L. (Eds.), Frontal lobe functioning and dysfunction (pp. 173–195). New York, NY: Oxford University Press.

[c26] Spreen O., & Strauss E. A. (1998). A compendium of neuropsychological tests: Administration, norms and commentary (2nd ed.). New York, NY: Oxford University Press.

[c27] Trenerry M. R., Crosson B., Deboe J., & Leber W. R. (1989). Stroop Neuropsychological Screening Test. Odessa, FL: Psychological Assessment Resources.

[c28] Turner M. S., Cipolotti L., Yousry T. A., & Shallice T. (2008). Confabulation: Damage to a specific inferior medial prefrontal system. Cortex: A Journal Devoted to the Study of the Nervous System and Behavior, 44, 637–648. 10.1016/j.cortex.2007.01.002 18472034

[c29] Warrington E. K., & James M. (1991). The Visual Object and Space Perception Battery. Bury St Edmunds, UK: Thames Valley Test Company.

[c30] Wheeler M. A., Stuss D. T., & Tulving E. (1995). Frontal lobe damage produces episodic memory impairment. Journal of the International Neuropsychological Society, 1, 525–536. 10.1017/S1355617700000655 9375239

